# Trips-Viz: a transcriptome browser for exploring Ribo-Seq data

**DOI:** 10.1093/nar/gky842

**Published:** 2018-09-20

**Authors:** Stephen J Kiniry, Patrick B F O’Connor, Audrey M Michel, Pavel V Baranov

**Affiliations:** School of Biochemistry and Cell Biology, University College Cork, Cork, Ireland

## Abstract

Ribosome profiling (Ribo-Seq) is a technique that allows for the isolation and sequencing of mRNA fragments protected from nuclease digestion by actively translating ribosomes. Mapping these ribosome footprints to a genome or transcriptome generates quantitative information on translated regions. To provide access to publicly available ribosome profiling data in the context of transcriptomes we developed Trips-Viz (t**r**anscriptome-wide **i**nformation on **p**rotein **s**ynthesis-v**i**suali**z**ed). Trips-Viz provides a large range of graphical tools for exploring global properties of translatomes and of individual transcripts. It enables analysis of aligned footprints to evaluate datasets quality, differential gene expression detection, visual identification of upstream ORFs and alternative proteoforms. Trips-Viz is available at https://trips.ucc.ie.

## INTRODUCTION

Ribosome profiling (1), also known as Ribo-Seq, is a technique that allows for large scale isolation of mRNA fragments that are being protected by actively translating ribosomes, see reviews (2–8). Sequencing these fragments, mapping them to a genome or transcriptome, and visualising these mappings can produce a global snapshot of which regions are being translated. There are a number of existing web based browsers which allow users to explore the alignments of publicly available ribosome profiling data. GWIPS-Viz (9) which provides both ribosome profiling and mRNA-seq data aligned to the genome was the first such browser developed for this purpose. To date, GWIPS-Viz hosts data from 23 organisms (10). SmProt (11) is another web based tool that aligns ribosome profiling data to the genomes of eight different organisms, combined with literature mining and mass spectrometry data it aims to find short translated ORFs (open reading frames) and allows users to explore each of these data types extensively. RPF-db (12) also permits visualisation of ribosome profiling data aligned to eight different organisms at a genomic level, as well as providing in depth information such as count tables, and meta-information such as the number of reads mapping to exonic/intronic/intergenic regions. Unlike these genome based tools, RiboViz (13) provides data aligned to the *Saccharomyces cerevisiae* transcriptome. It processes the data to analyse useful characteristics of the datasets, e.g. readlength distribution, triplet periodicity, as well as translation efficiencies. TranslatomeDb also aligns Ribo-Seq data to the transcriptomes of 13 different organisms, along with RNA-Seq and RNC-Seq data (14).

Mapping data to the transcriptome has certain advantages over mapping to the genome. Ribo-Seq reads are typically short (∼30 nucleotides in length) and so the difficulty of mapping these short reads across splice junctions is relieved. The absence of long or numerous intronic regions makes the interpretation of the mapped reads easier from a user perspective when mapping to a transcriptome. However, it should be noted that aligning to the transcriptome is not inherently superior to genomic alignments, transcriptomic alignments for example are annotation dependent, meaning alignments would have to be re-done for each different version of the transcriptome. Transcriptome aligned data cannot be used for the analysis of translation outside of exons, e.g. translation of retained introns (15). As both methods have their advantages/disadvantages it would be best to make use of both transcriptomic and genomic alignments when analysing sequencing data.

Trips-Viz presents transcriptomic alignments of Ribo-Seq and mRNA-seq data. Currently the number of organisms available in Trips-Viz stands at 7 (*Homo sapiens, Rattus norvegicus, Saccharomyces cerevisiae, Mus musculus, Drosophila melanogaster, Escherichia coli* and *Caenorhabditis elegans*). At the time of writing there are 1460 Ribo-Seq datasets and 335 mRNA-seq datasets available.

Trips-Viz utilizes a number of visualization solutions, not implemented by other tools. For instance, reads are coloured depending on matching subcodon position, to visualize triplet periodicity of Ribo-Seq data. Colour coding the reads can give a clear picture of which reading frames of a transcript are likely being translated, particularly if using an aggregate of data from many studies. This is particularly useful when multiple ORFs of the same transcript are being translated, e.g. CDS (Coding Sequence) and overlapping upstream ORFs (16).

Trips-Viz provides a versatile set of graphical analysis tools including the readlength distribution, triplet periodicity, metagene profiles and more. Trips-Viz also provides the ability to plot multiple datasets on the same graph for the same transcript. This allows for comparison of translated features across different samples, e.g. cell lines/tissues as well as across conditions and in response to drug treatments. Lastly, Trips-Viz allows the user to detect differentially expressed genes (at the level of RNA and protein synthesis).

## MATERIALS AND METHODS

The Trips-Viz pipeline for processing Ribo-Seq data is as follows: publicly available ribosome profiling and corresponding RNA-seq datasets are downloaded from the gene expression omnibus https://www.ncbi.nlm.nih.gov/sra/ in SRA format. These are converted to FASTQ format and then the adapter sequence is clipped using cutadapt (18), reads below 25 nucleotides are removed. Bowtie (19) is then used to remove any reads mapping to ribosomal RNA. Bowtie is again used to map the remaining reads to a reference transcriptome. Samtools (20) is used to convert the resulting SAM file to BAM file format. Finally, the BAM file is parsed using a custom python script to pull out the necessary information for Trips-Viz, this includes determination of offsets for Ribo-Seq reads. This is a numerical value added to the position of the 5′ end of reads (or subtracted from the 3′ end) to approximate the A-site. This is done by creating a metagene profile, an aggregation of reads from all coding transcripts centred around annotated start codons. The distance in nucleotides between the highest peak upstream of the start codon (or downstream if determining a 3′ end offset) and the start codon itself (located at the P-site) is determined. This value is modified by adding 3 to set the 5′ end offset (or subtracting 3 to set the 3′ offset). Both 5′ end offsets and 3′ end offsets are determined separately for every read length. Offsets and other information extracted from the BAM file are stored in SQLite format.

The web framework for Trips-Viz is handled using the python package Flask (http://flask.pocoo.org/). All plots are generated using either mpld3 (http://mpld3.github.io) or bokeh (https://bokeh.pydata.org/en/latest) python packages. Currently we intend to include all publicly available Ribo-Seq data, however this may change as the number of ribosome profiling studies increases.

## DISCUSSION

The primary use of Trips-Viz is the interactive visualization of an aggregate of ribosome profiling data at subcodon resolution in the context of single transcripts, a feature not provided by other existing databases. To do this the user selects an organism and transcriptome assembly and then selects ***Single transcript plot***. Settings such as the gene of interest, minimum and maximum readlengths, ambiguous mapping filters and other settings can be changed at the top of the page. Ribo-Seq and mRNA-seq data files can be chosen at the centre of the page by selecting a sequence type, a study name and then clicking checkboxes next to file names. Clicking the *View Plot* button at the end of the page will produce a plot of the transcript in question. More detailed instructions on how to select data files and what each setting does can be found on the help pages or by clicking the link next to any of the settings labelled ‘*What's this*’.

There are three horizontal bars below the plot coloured in red, green and blue. These represent the three reading frames of the transcript, with short vertical white lines representing start codons and longer vertical grey lines representing stop codons. The main window shows densities of mapped footprints as line graphs of either red, green, or blue colors depending on the reading frame whose translation is the best supported by the reads based on their alignments relative to subcodon positions. The colored boxes on the right of the graph represent the control panel with colored buttons that allow the user to hide/display corresponding items in the main window. There are four icons below the plot, the first three when clicked, allows users to reset/move/zoom the view in the main window. The fourth icon allows the user to download the nucleotides sequence and read counts from the current transcript in .csv format.

**Figure 1.**
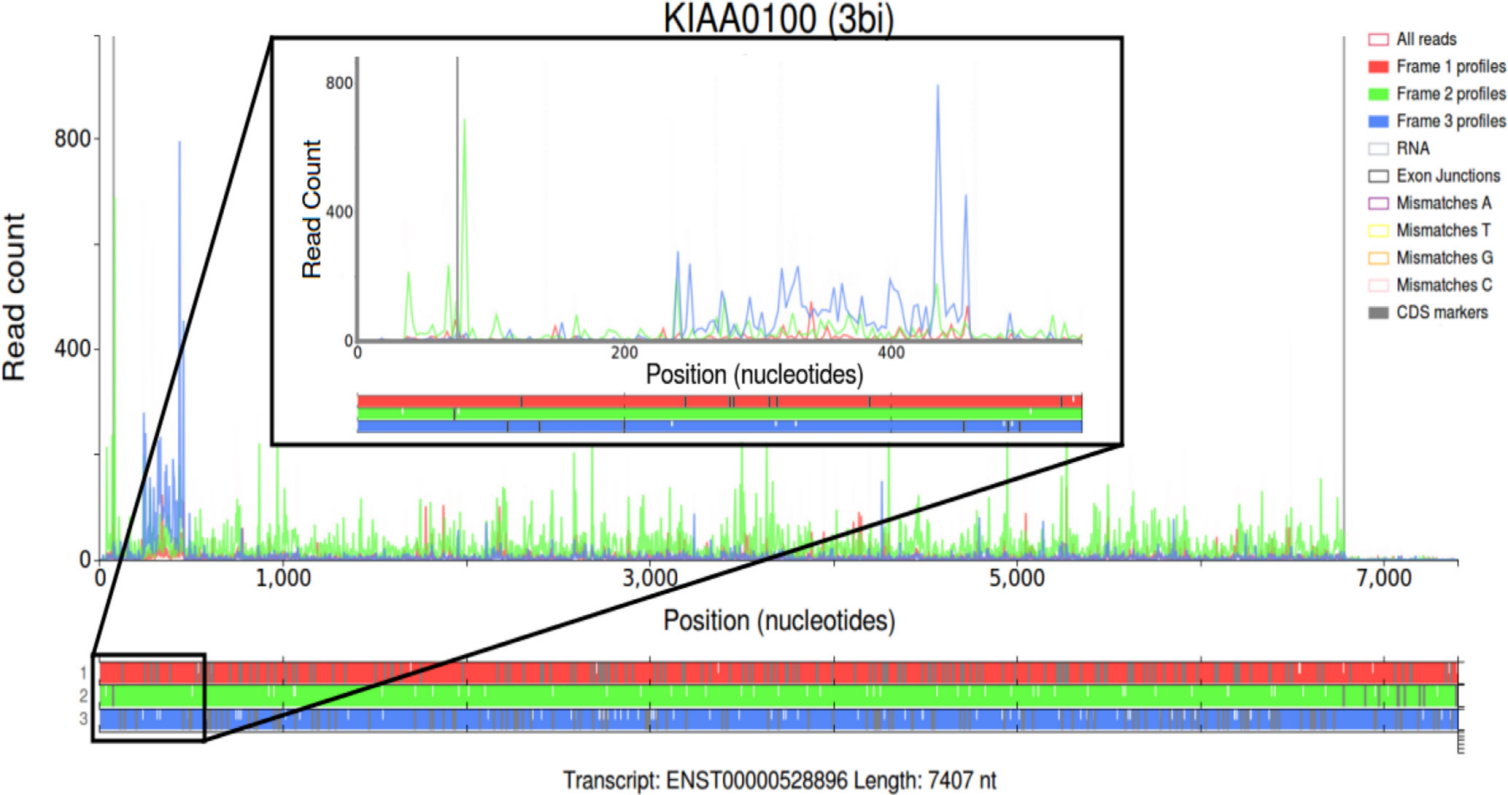
Modified screenshots of the Trips-Viz single transcript plots for a Gencode transcript of the human *KIAA0100* gene (large plot) and its 5′ area (small plot). Ribo-Seq read densities are displayed in the main window, color coded according to their mapping phase relative to the reading frame subcodon positions. Transcript coordinates are shown on the x-axis, while read counts are shown on the y-axis. The ORF architecture is shown below with three different reading frames differentially colored, stop codons indicated as vertical grey dashes and AUGs as white dashes.

To demonstrate the utility of this plot an example is shown in Figure 1. Here a plot from the s*ingle transcript plot* page of Trips-Viz has been generated for the human *KIAA0100* gene using an aggregate of Ribosome Profiling datasets. The annotated coding region of this transcript starts at position 76 in the second frame (green). As can be seen in the figure most of the Ribo-Seq reads after position 76 are represented predominantly by green line graphs (up until the annotated stop codon at position 6781 where the read density decreases drastically) indicating translation in the second frame, as expected. Translation of a short upstream ORF at the coordinates 34–73 is also evident. Within the CDS one notable exception to the predominantly green reads lies between positions 236 and 455 where the reads are predominantly blue. This corresponds to an ORF within the third line (blue) of the ORF architecture, which likely means this ORF is also translated. Detection of such nested ORFs in particular highlights the currently unique utility of Trips-Viz that is enabled by differential read density colouring.

Another useful feature of Trips-Viz is the ability to plot data obtained from multiple different samples on the same transcript simultaneously to allow comparative analysis. This can be achieved using the *single transcript comparison plot*. Here users can specify the transcript at the top of the page and choose whether to normalize the data over the number of mapped reads per sample, which is useful when comparing datasets with large differences in coverage. Users can set up groups of data using study names at the center of the page. This is done by selecting a colour (by clicking on the colored button), selecting a file and then clicking the *Add* button. The data between the groups are differentially colored enabling comparison *via* visual inspection.

**Figure 2.**
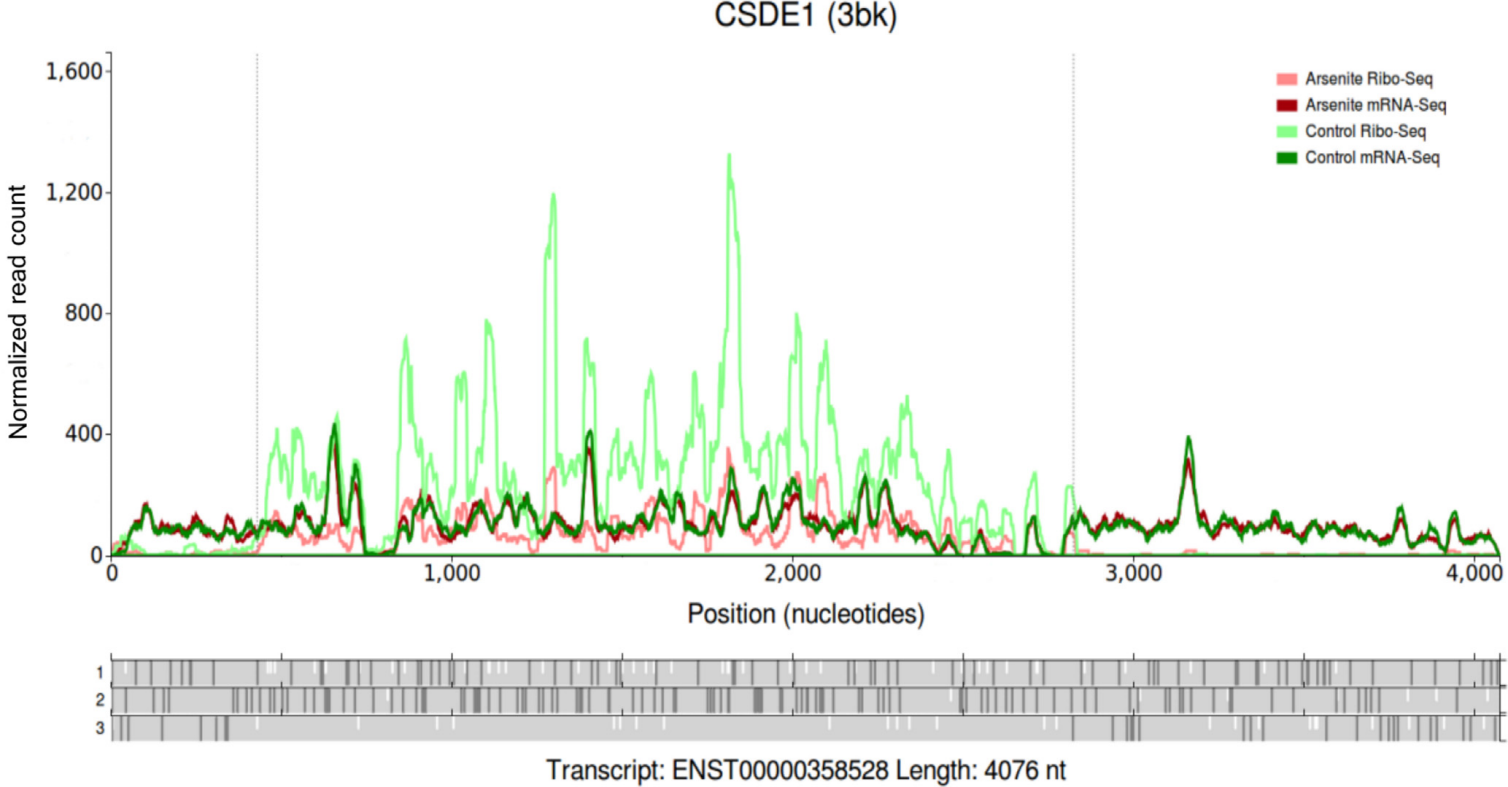
A modified screenshot of a single transcript comparison plot for *CSDE1* gene. The read densities from four datasets are shown as line graphs highlighted differentially as indicated by the legend in the top right corner. The other features are similar to Figure 1.

An example is shown in Figure 2 for the human *CSDE1* that illustrates how its translation is changed during Integrated Stress Response (ISR) using data from the Andreev *et al.*'s study (17). For the samples treated with sodium arsenite (a trigger of ISR), Ribo-Seq and RNA-Seq read densities are displayed using line graphs of light red and dark red colours respectively. Read densities from untreated control samples are displayed in light green (Ribo-Seq) and dark green (RNA-Seq). It can be seen that both mRNA-Seq datasets have very similar densities, indicating that there is little or no RNA level changes in response to the arsenite treatment. In contrast, the Ribo-Seq density from arsenite treated cells is lower than that for the Ribo-Seq data obtained from the untreated cells, indicating that translation of this gene is reduced substantially during ISR in comparison with translation of other genes.

**Figure 3.**
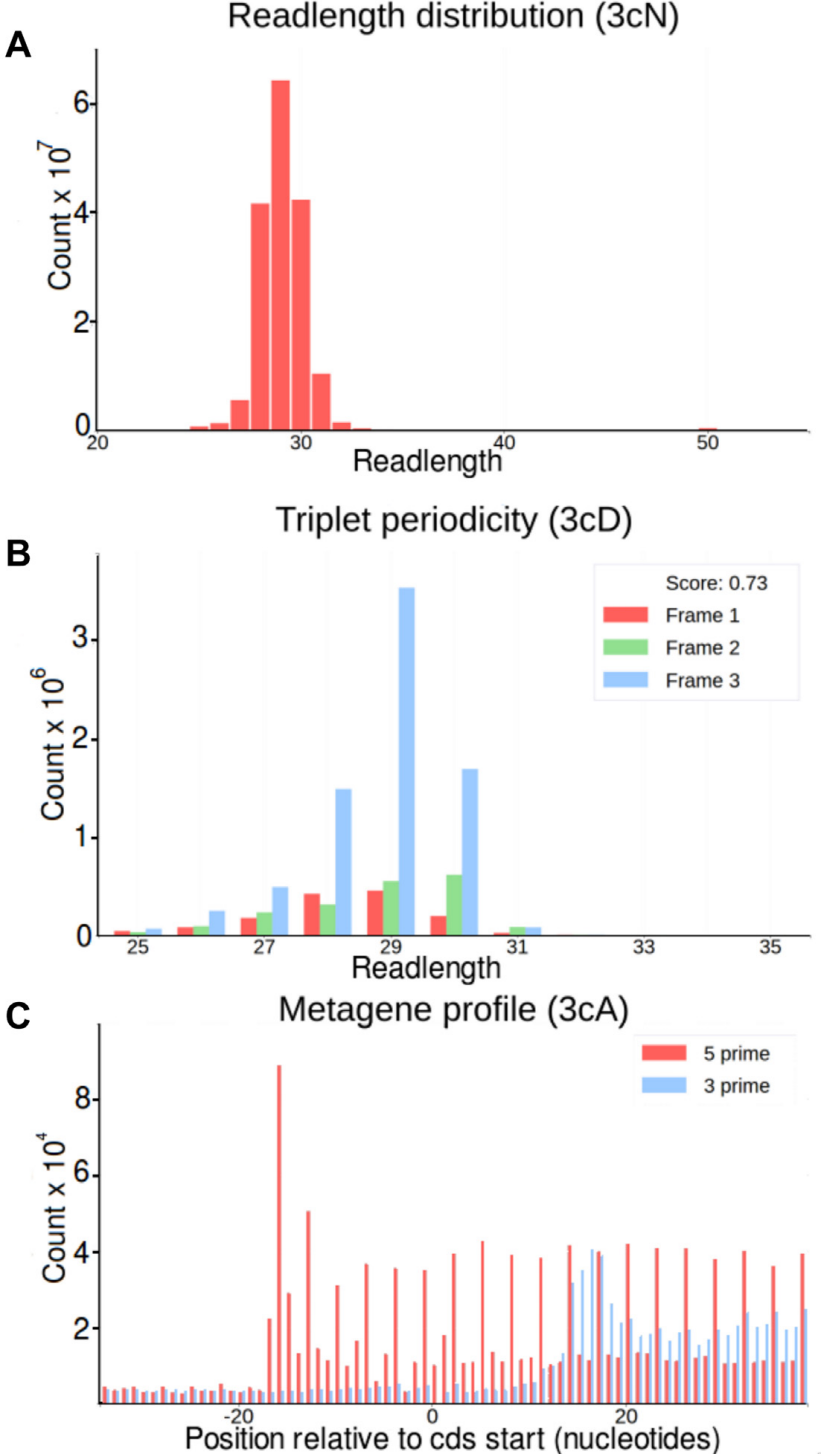
Dataset characterizations. (**A**) Distribution of read lengths from Matsuo *et al.* dataset (23). (**B**) Triplet periodicity plot for a Ribo-Seq dataset from Loayza-Puch *et al.* (24). Here each readlength is displayed using 3 bars depending on their phase to the first subcodon position of three different reading frames. Only reads aligned to annotated coding regions are used in this plot. The difference between bars indicates the strength of triplet periodicity. The datasets with stronger periodicity has a greater power for detecting translated reading frames as in the example shown in Figure 1. (**C**) A metagene profile of a Ribo-Seq dataset from Neri *et al.* (25). Here, the frequency of Ribo-Seq reads is shown relative to start codons (0 coordinate) across all protein coding transcripts and displayed either for reads 5′ (red) or 3′ (blue) ends. Since most ribosome footprints are expected to be found inside CDS regions, an increase in ribosome density is expected upstream of CDS. Metagene plots can be used for inferring an offsets between the decoding center of the ribosome (A or P-sites) and the ends of ribosome footprints. The plot also indicates the strength and consistency of triplet periodicity.

Unlike the two previous plot types the *meta-information* page gets its information from an entire dataset, aggregating information from multiple transcripts, for example, the triplet periodicity plot displays information from all annotated coding transcripts. This page allows the user to create a number of different plots which can be selected at the top left of the page. File selection is handled at the center of the page in the same manner as the *single transcript plot* page. In general, this page can be used to assess the quality of datasets as these plots provide general characteristics of the datasets that could reveal dataset defects. Examples are shown in Figure 3. A detailed description of each plot type can be found on the help pages, https://trips.ucc.ie/help.

**Figure 4.**
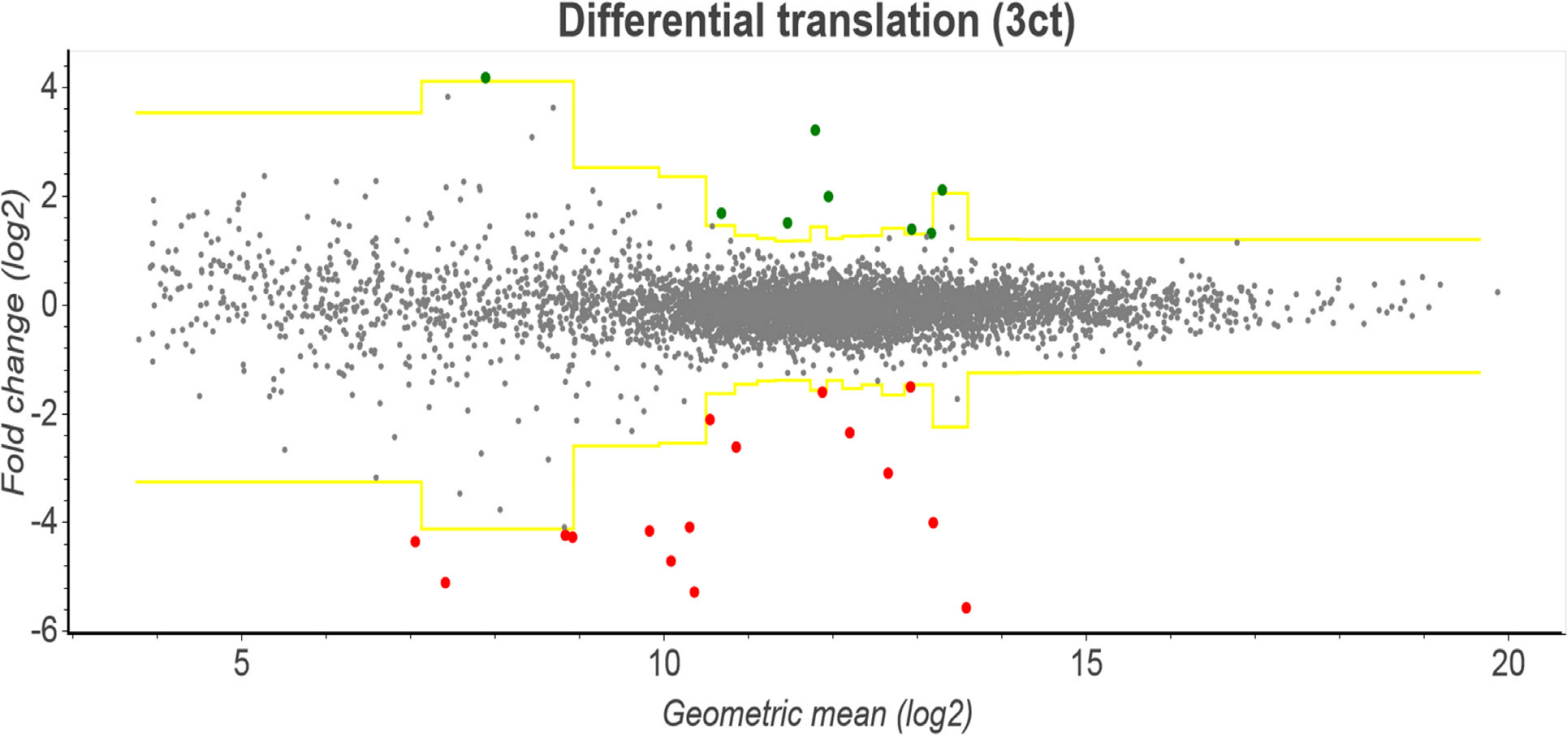
A modified screenshot of Trips-Viz showing a plot from the Differential plot page for the datasets obtained in the Albert *et al.* dataset (26). Here, fold change log ratios are shown on the y-axis while the geometric mean of the read counts in each condition is shown on the x-axis. Transcripts are grouped into bins of size 300 based on the geometric mean. Based on parameters of log ratios within each bin, a *z*-score is calculated for each transcript. The yellow lines on this graph represent the positive and negative *z*-score threshold (as chosen by the user), and transcripts that fall above/below that threshold are colored green/red.

Lastly there is the *differential plot* page, where users can find genes whose expression is significantly up/down-regulated relative to others. Users can organize the data into groups and compare relative RNA levels or protein synthesis levels between the groups and set minimum/maximum *z*-scores at the top of the page. Up/down-regulated transcripts will then be detected using the *z*-score transformation approach (17). An example of the resulting plot can be seen in Figure 4. Here transcripts are represented as points on a scatter plot, with yellow lines specifying the upper and lower thresholds to indicate the *z*-score cut-off (as chosen by the user). Points above the upper threshold are colored green (up-regulated) while points below the lower threshold are colored red (down-regulated). Hovering the mouse cursor over a specific point will display the transcript ID and the number of reads mapped to it, while clicking on the point will open up a separate tab where the read densities for that gene will be displayed on the *single transcript comparison plot* page.

In addition to data visualizations Trips-Viz provides a platform for collaborative research and data sharing. For every plot created on Trips-Viz a URL is created which contains information such as the files and settings used to create the plot. This URL can then be sent to another user, where trips will use the information in the URL to recreate the plot in their browser. For convenience, rather than displaying the URL directly to the user, the URL is given a unique short code which is visible between parentheses in the title of every plot on Trips-Viz, including the plots presented in this manuscript. The URL can then be sent in the following form https://trips.ucc.ie/short/short_code. For example to recreate the plot shown in Figure 1 users can follow the link https://trips.ucc.ie/short/3bi and explore the plot interactively in a browser. These links will last for the lifetime of Trips-Viz, with the exception of links associated with private data.

Private data can be uploaded by any user with an account on Trips-Viz, an account can be created using the *Sign up* link at the top of any page. Uploaded data must be in a specific format which can be created by running a python script and passing it a BAM file. Users can download this script from the Trips-Viz *downloads* page, a link to which is given at the top of every page and instructions on how to use it are included in the script itself. The *downloads* page also provides the relevant transcriptome fasta file and gtf file for each organism/assembly in Trips-Viz. Files can be uploaded using the *uploads* link at the top of every page. The user's data will be securely hidden from all other users by default but the uploader can share the data with other users of their choosing *via* the *uploads* page. Signing up also allows users to customize the graphic display of Trips-Viz, e.g. the background colour of plots. This can be accessed by visiting the *settings* link at the top of any page while signed in.

We plan to continually expand the number of organisms and Ribo-Seq/mRNA-Seq datasets available in Trips-Viz by including data as they become publicly available. However, it is conceivable that our computational capacities will not match the rapid pace of data growth. In this case we aim to develop a policy for data selection/prioritization based on data quality and their general scientific interest. We plan to streamline uploading of private data by providing a data processing workflow on Ribogalaxy (21). We also plan to generate a docker image of the site for users who may want to run their own instance of Trips-Viz. Also, we intend to explore the possibility of providing other types of publicly available sequencing data that are relevant to mRNA translation, e.g. epitranscriptomics data (22). We encourage users to contact us *via* the contact page https://trips.ucc.ie/contactus to provide feedback or suggestions, Trips-Viz related comments are also welcomed at the GWIPS-viz forum https://gwips.ucc.ie/Forum/. The current version of Trips-Viz was optimized and tested with Chrome and Firefox browsers. Its full functionality with other Internet browsers is not guaranteed at present.
